# PKCε-dependent potentiation of TTX-resistant Na_v_1.8 current by neurokinin-1 receptor activation in rat dorsal root ganglion neurons

**DOI:** 10.1186/1744-8069-5-33

**Published:** 2009-06-30

**Authors:** Chun-Lei Cang, Hua Zhang, Yu-Qiu Zhang, Zhi-Qi Zhao

**Affiliations:** 1 Institute of Neurobiology, Institutes of Brain Science and State Key Laboratory of Medical Neurobiology, Fudan University, Shanghai 200032, PR China

## Abstract

**Background:**

Substance P (SP), which mainly exists in a subtype of small-diameter dorsal root ganglion (DRG) neurons, is an important signal molecule in pain processing in the spinal cord. Our previous results have proved the expression of SP receptor neurokinin-1 (NK-1) on DRG neurons and its interaction with transient receptor potential vanilloid 1 (TRPV1) receptor.

**Results:**

In this study we investigated the effect of NK-1 receptor agonist on Na_v_1.8, a tetrodotoxin (TTX)-resistant sodium channel, in rat small-diameter DRG neurons employing whole-cell patch clamp recordings. NK-1 agonist [Sar^9^, Met(O_2_)^11^]-substance P (Sar-SP) significantly enhanced the Na_v_1.8 currents in a subgroup of small-diameter DRG neurons under both the normal and inflammatory situation, and the enhancement was blocked by NK-1 antagonist Win51708 and protein kinase C (PKC) inhibitor bisindolylmaleimide (BIM), but not the protein kinase A (PKA) inhibitor H89. In particular, the inhibitor of PKCε, a PKC isoform, completely blocked this effect. Under current clamp model, Sar-SP reduced the amount of current required to evoke action potentials and increased the firing rate in a subgroup of DRG neurons.

**Conclusion:**

These data suggest that activation of NK-1 receptor potentiates Na_v_1.8 sodium current via PKCε-dependent signaling pathway, probably participating in the generation of inflammatory hyperalgesia.

## Background

Substance P (SP), a member of tachykinin family, is a well-known pain-related neuropeptide in the spinal cord. It is released by unmyelinated primary afferent fiber terminals of small-diameter dorsal root ganglion (DRG) neurons and participates in the spinal transmission of nociceptive signals [1-3]. It is well documented that the SP receptor neurokinin-1 (NK-1) is densely distributed in the superficial dorsal horn and involved in the development of chronic pain and central sensitization after intense noxious stimulation and tissue/nerve injury [4-7].

In addition to the expression of the NK-1 on the postsynaptic neurons of superficial spinal dorsal horn, increasing evidence strongly suggested the presynaptic expression of NK-1 in DRG neurons. The immunohistochemical evidence revealed that the NK-1 was expressed by the unmyelinated axons of the glabrous skin [8], and the DRG neuron soma in rats [9]. By means of intracellular and whole-cell patch clamp recordings, SP was shown to be able to induce the depolarization of DRG or trigeminal ganglion neurons in the different species [10-13] and potentiated the TRPV1 currents [9]. However, the function of DRG-expressed NK-1 receptor needs to be further understood.

The Na_v_1.8, which is a TTX-resistant sodium channel and mainly expressed in small-diameter DRG neurons [14,15], is a major contributor to the upstroke of action potential in these neurons [16]. In the Na_v_1.8-null mice or Na_v_1.8 knockdown mice by antisense oligodeoxynucleotides, both the physiological and pathological pain was alleviated [17-20]. Accumulative evidence showed that the Na_v_1.8 current was regulated by various inflammatory mediators, such as prostaglandin E_2 _(PGE_2_), serotonin, NGF etc. through a PKA or PKC signaling pathway [21-24]. 

In the present study, we investigated the effects of the NK-1 agonist on dynamics of Na_v_1.8 currents in isolated small-diameter DRG neurons using whole-cell patch clamp recording. Also, the role of PKC signal pathway in the cross-talk between NK-1 and Na_v_1.8 was examined.

## Results

### Recording of Na_v_1.8 currents in DRG neurons

**Figure 1:**
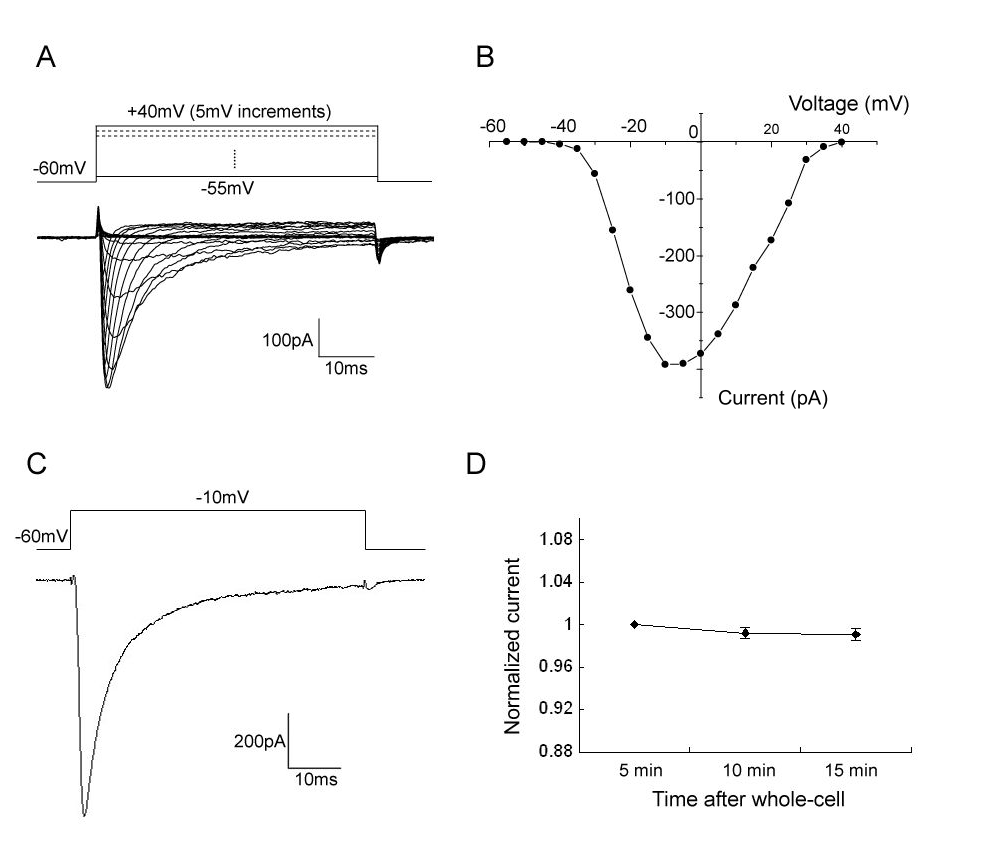
**Recording of Na_v_1.8 currents in rat DRG neurons**. A: representative I-V curve family of currents recorded in the presence of 500 nM TTX is shown, using a protocol (inset) where cells were depolarized to a variety of potentials (-55 to +40 mV) from a holding potential of -60 mV to elicit Na_v_1.8 currents. B: *I-V *curve of Na_v_1.8 currents shown in (A). C: representative traces of Na_v_1.8 current elicited by a single pulse of -10 mV which was used in most of the recordings. D: peak amplitudes of Na_v_1.8 currents elicited by -10 mV pulse at 5 min, 10 min and 15 min after whole-cell mode was performed. The currents were stable during the recording time in all the cells (n = 16).

With existence of TTX (500 nM) in external solution, TTX-resistant sodium currents were recorded in most (166 out of 205) of the small-diameter DRG neurons (<25 μm). The membrane potential was hold to -60 mV. Under this recording condition, TTX-resistant sodium currents were mainly mediated by Na_v_1.8 channels due to inactivation of Na_v_1.9 [25,26]. The family of Na_v_1.8 sodium currents was generated with a voltage-clamp protocol (depolarizing steps from -55 mV to 40 mV, 50 ms, 5 mV increments, Figure 1A). In accordance with the current-voltage relationship (Figure 1B), -10 mV was chosen to elicit Na_v_1.8 currents in most of the recordings (Figure 1C). As reported by Saab et al. [27], fluoride-based pipette solution also caused slow stabilization of the amplitude of Na_v_1.8 current after rupture of cell membrane in our experiments. We measured the peak amplitude of the Na_v_1.8 current at 5 min, 10 min and 15 min after whole-cell mode was performed. As shown in Figure 1D, the peak amplitude was relatively stable from 5 min to 15 min (n = 16). All of our subsequent recordings were performed in this time course.

### Increase in peak amplitude of Na_v_1.8 current by Sar-SP

**Figure 2:**
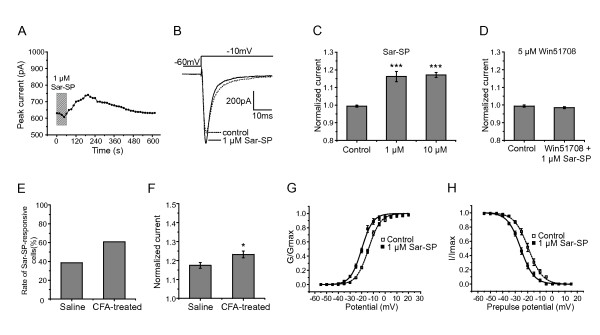
**Effect of NK-1 agonist Sar-SP on Na_v_1.8 currents**. A: time course of the potentiation effect of Sar-SP. The maximal increase in the peak amplitude was at 3 min after Sar-SP perfusion, and reduced slowly to control level thereafter. B: typical traces illustrating the Na_v_1.8 current recorded in a neuron pre- (dashed line, control) and post- (solid line, Sar-SP) perfusion of 1 μM Sar-SP. C: histogram showing the effect of 1 μM and 10 μM Sar-SP. The normalized peak current was enhanced to 116.2 ± 2.9% and 117.1 ± 1.4% 3 min after perfusion of 1 μM and 10 μM Sar-SP, respectively (***p < 0.001, versus control, Kruskal-Wallis one-way ANOVA, n = 16 for control, 13 for 1 μM, and 6 for 10 μM). D: NK-1 antagonist Win51708 (5 μM) completely blocked the effect of Sar-SP in all 15 neurons tested (*p *> 0.05, t-test). E: The rate of the Sar-SP-responsive cells was increased after CFA-treatment. F: The effect of Sar-SP was also increased after peripheral CFA-treatment (* *p *< 0.05, t-test, n = 8 for saline and 14 for CFA-treated). G and H: Sar-SP shifted the activation (G) and steady-state inactivation (H) curve in a hyperpolarizing direction.

Following the perfusion with Sar-SP (1 μM, 1 min), a selective NK-1 agonist, Na_v_1.8 currents were increased in 13 out of 30 DRG neurons tested (Figure 2A, B and 2C). The maximal enhancement of the peak amplitude occurred at 3 min after Sar-SP perfusion, and reduced slowly to control level thereafter (Figure 2A). As shown in Figure 2C, normalized currents were increased significantly by Sar-SP (116.2 ± 2.9%, n = 13) compared with the control (99.2 ± 0.5%, n = 16, *p *< 0.001). Higher concentration of Sar-SP (10 μM) failed to induce more powerful action (117.1 ± 1.4%, n = 6, *p *< 0.001, Figure 2C), suggesting a "ceiling effect" at 1 μM. The Sar-SP-induced potentiation was blocked by co-incubation of Win51708, a selective NK-1 antagonist (5 μM, 98.5 ± 0.6%, n = 15, Figure 2D). To exclude the influence of fluoride, we also test the effect of Sar-SP by using chloride-based pipette solution. Cesium fluoride was changed to cesium chloride. The effect of Sar-SP wasn't changed significantly (data not shown).

The effect of Sar-SP on Na_v_1.8 current was also examined under peripheral inflammation condition. Complete Freund's adjuvant (CFA, 100 μl) was bilaterally injected into rat hind paws. DRG neurons was tested three days after CFA treatment, Na_v_1.8 currents were enhanced by Sar-SP (1 μM) in 60.9% of DRG neurons (n = 23). In the saline treated control rats, 38.1% of DRG neurons (n = 21) exhibited potentiation of Na_v_1.8 current (Figure 2E). In rats with CFA treatment, the peak Na_v_1.8 current in DRG neurons was enhanced to 123.2 ± 1.8% (n = 14) following application of Sar-SP, whereas enhanced to 117.4 ± 1.5% (n = 8) in saline treated control rats (Figure 2F, *p *< 0.05). Given inflammation-induced increase in expression of NK-1 [9], it is suggested that such modulation of NK-1 may be more beneficial for controlling inflammatory chronic pain.

### Sar-SP shifted the activation and steady-state inactivation curves of Na_v_1.8 in a hyperpolarizing direction

As described above, a voltage-clamp protocol consisted of 50 ms depolarizing steps from -55 mV to 40 mV (5 mV increments) was used to determine the activation of Na_v_1.8 channels. Sar-SP caused a left shift of the activation curve (Figure 2G). Half-maximal activation potential (V_1/2activation_) was shifted to -20.03 ± 0.23 mV from that of control condition (-14.24 ± 0.25, n = 6) after Sar-SP perfusion. The *k *value was not changed by Sar-SP (*k*_control _= 4.89 ± 0.27, *k*_Sar-SP _= 4.77 ± 0.45). Steady-state inactivation was determined at a series of membrane potentials from -60 mV to 15 mV (5 mV increments) for 500 ms and a following test potential of 20 mV. Similar to the activation curve, the steady-state inactivation curve was also shifted in a hyperpolarized direction after Sar-SP perfusion (Figure 2H). The V_1/2 _of voltage dependence of steady-state inactivation was -19.31 ± 0.23 mV (n = 6) using vehicle alone, and -25.55 ± 0.14 mV after Sar-SP treatment (n = 6). The *k *value was also not changed by Sar-SP (*k*_control _= 5.39 ± 0.37, *k*_Sar-SP _= 5.09 ± 0.32).

### Sar-SP-induced potentiation of Na_v_1.8 via PKC, but not PKA

**Figure 3:**
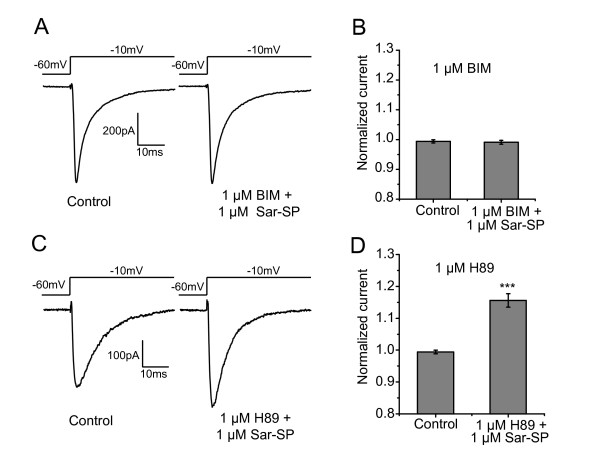
**Involvement of PKC, but not PKA in Sar-SP-induced potentiation of Na_v_1.8 currents**. Incubation with PKC inhibitor BIM (1 μM) for 30 min before Sar-SP perfusion completely blocked the potentiation effect of Sar-SP in all 24 neurons tested (A and B, *p *> 0.05, Mann-Whitney rank sum test). After incubation with PKA inhibitor H89, Sar-SP (1 μM) still fully enhanced Na_v_1.8 currents in 9 of 24 DRG neurons (C and D, ***p < 0.001, Mann-Whitney rank sum test).

Activation of NK-1, a G-protein coupled receptor, triggers several intracellular signal pathways, such as PKA and PKC pathway [28]. Given modulation of Na_v_1.8 by PKC [23,24,29], here we used bisindolylmaleimide (BIM), a PKC inhibitor, to examine its effect on the Sar-SP-induced potentiation of Na_v_1.8 currents. In all DRG neurons (n = 24) incubated with BIM (1 μM) for 30 min before Sar-SP perfusion, Sar-SP failed to potentiate Na_v_1.8 currents, suggesting a PKC-dependent mechanism in the interaction of NK-1 and Na_v_1.8 (Figure 3A and 3B).

Na_v_1.8 currents were also reported to be regulated by PKA [22,23,30]. While the cells were incubated with the PKA inhibitor H89 (1 μM) for 30 min, Sar-SP (1 μM) still fully enhanced Na_v_1.8 currents in 9 of 24 DRG neurons (Figure 3C and 3D, control = 99.2 ± 0.5%, Sar-SP = 115.6 ± 2.1%, *p *< 0.001), suggesting that the PKA pathway did not participate in the interaction of NK-1 and Na_v_1.8.

**Figure 4:**
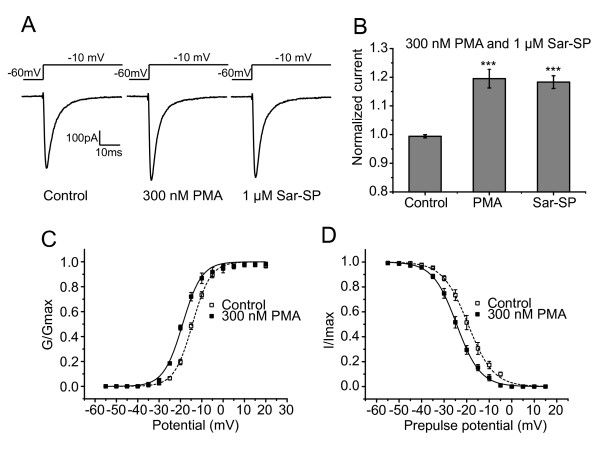
**PMA mimic the effect of Sar-SP**. A and B:representative traces (A) and histogram (B) showing the effect of PMA on Na_v_1.8 currents. 300 nM PMA induced a similar potentiation to 1 μM Sar-SP. Perfusion with Sar-SP failed to further enhance the currents after PMA-induced peak potentiation (***p < 0.001, versus control, Kruskal-Wallis one-way ANOVA, n = 16 for control, 10 for PMA and 10 for Sar-SP). PMA also shifted the activation and steady-state inactivation curve of Na_v_1.8 in a hyperpolarized direction (C and D).

Further, PMA (phorbol 12-myristate 13-acetate), a PKC activator, was used to mimic the effect of Sar-SP. As shown in Figure 4, PMA (300 nM) significantly enhanced the Na_v_1.8 currents by 19.5 ± 3.2% in 10 out of 13 recorded neurons (*p *< 0.001, Figure 4A and 4B). Notably, perfusion with Sar-SP (1 μM) failed to further enhance PMA-induced potentiation of Na_v_1.8 currents (118.3 ± 2.2%, *p *< 0.001, Figure 4A and 4B). After PMA perfusion, the activation and steady-state inactivation curves were also shifted in a hyperpolarizing direction (Figure 4C and 4D). Half-maximal activation potential (V_1/2 activation_) was shifted to -18.97 ± 0.28 mV from that of control condition (-14.37 ± 0.18 mV, n = 6). The half-maximal inactivation potential (V_1/2 inactivation_) was shifted to -24.48 ± 0.10 mV from -19.41 ± 0.18 mV (control, n = 6). These results further confirmed that Sar-SP modulated Na_v_1.8 channels in a PKC-dependent pathway. In consistent with Sar-SP, PMA didn't change the *k *value in the activation and inactivation curves (activation: *k*_control _= 4.59 ± 0.26, *k*_PMA _= 4.64 ± 0.68; inactivation: *k*_control _= 5.39 ± 0.37, *k*_PMA _= 5.14 ± 0.36).

### PKCε: a pivotal factor for potentiation of Na_v_1.8 by NK-1 activation

**Figure 5:**
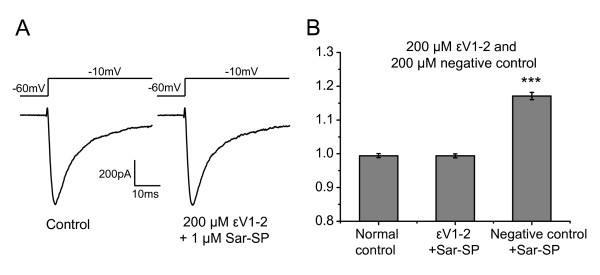
**PKCε was the main PKC subtype mediating the effect of Sar-SP**. A and B: representative traces (A) and histogram (B) showing the effect of PKCε inhibitor εV1-2 on Sar-SP-induced potentiation. Intracellular application of εV1-2 (200 μM) completely abolished the potentiation effect of Sar-SP. The negative control (control peptide) of εV1-2 failed to block Sar-SP-induced potentiation (***p < 0.001, versus normal control, one-way ANOVA, n = 16 for normal control, 11 for εV1-2 and 8 for negative control).

As reported, there are five isoforms of PKC expressed in DRG neurons [31]. Among these isoforms, PKCε is highly expressed in small-diameter DRG neurons and involved in NK-1 activation-induced potentiation of TRPV1 and the development of hyperalgesia [9,31-33]. Therefore, to explore the effect of PKCε on Sar-SP-induced potentiation of Na_v_1.8, εV1-2 (200 μM), a specific PKCε inhibitor, was delivered intracellularly via recording electrodes. The potentiation of Na_v_1.8 currents was completely blocked by εV1-2 (n = 15), but not by its negative control (control peptide) (n = 8, Figure 5A and 5B).

### Effect of Sar-SP on excitability of DRG neuron

**Figure 6:**
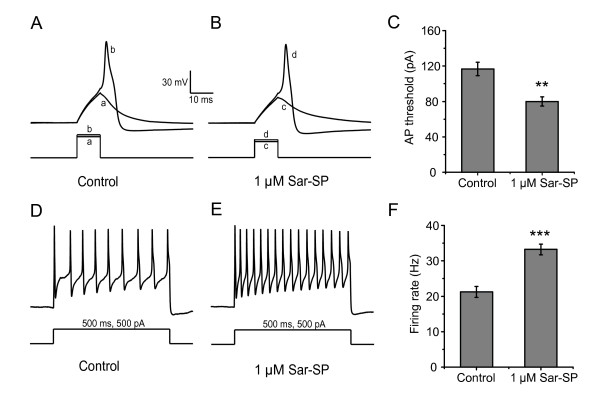
**Effect of 1 μM Sar-SP on action potential threshold and firing rate in DRG neurons**. Sar-SP reduced the amount of current required to evoke action potential and increased the firing rate in DRG neurons. Experiments were performed using current clamp model. A and B: depolarizing current pulse required to evoke an action potential in a DRG neuron, before (A) and after (B) application of Sar-SP (a = 130 pA, b = 140 pA, c = 80 pA, d = 90 pA). C: effect of Sar-SP on the threshold for action potential generation by depolarizing current pulse (**p < 0.01, paired t-test, n = 6). D and E: firing response of a DRG neuron to a 500 pA depolarizing current pulse (500 ms), before (D) and after (E) application of Sar-SP. F: effect of Sar-SP on firing rate in DRG neurons (***p < 0.001, paired t-test, n = 6).

Na_v_1.8 is the main contributor to the upstroke of action potentials in small-diameter DRG neurons [16]. Therefore, modulation of this channel by Sar-SP should influence excitability of the DRG neuron. We detected the effect of Sar-SP on the threshold for evoking action potential in DRG neurons. To evoke action potentials, 5 ms step depolarizing current pulses were applied to neurons before and after exposure to Sar-SP (Figure 6A and 6B). In 6 of 17 neurons tested, Sar-SP significantly decreased the injected current threshold to evoke action potentials. Under the control condition, the current threshold was 116.7 ± 7.6 pA. After exposure to Sar-SP, this threshold was reduced to 80.0 ± 5.2 pA (Figure 6C, n = 6, *p *< 0.01).

In addition, during injection of supramaximal depolarizing current pulses (500 ms, 500 pA), DRG neurons fired with a frequency at 21.22 ± 1.57 Hz (Figure 6D and 6F, n = 6). Sar-SP significantly increased the firing frequency to 33.22 ± 1.53 Hz (Figure 6E and 6F, n = 6, *p *< 0.001).

## Discussion

The role of substance P (SP) and its NK-1 receptor in pain processing was widely investigated in the spinal cord. However, most of the prevailing studies focused on the postsynaptic NK-1 receptors in the spinal superficial dorsal horn neurons. Whether NK-1 receptors are also expressed presynaptically in primary sensory neurons is still obscure. A growing body of evidence showed that SP could activate DRG neurons through NK-1 receptor in primary sensory neurons [10-13,34,35], in despite of a contradictory report [36]. Our recent study provided new evidence for the expression of NK-1 receptor protein and interaction with TRPV1, a crucial pain signal molecule, in DRG neurons [9].

In addition to TRPV1, another important ion channel, TTX-resistant sodium channel, is also primarily expressed in nociceptors. Between the two distinct TTX-resistant sodium channel isoforms Na_v_1.8 and Na_v_1.9, Na_v_1.8 likely mediates the majority of the TTX-resistant currents and plays an important role in pain processing. Na_v_1.8-null mice displayed a pronounced increase in threshold to noxious mechanical stimuli and a slight decrease in nociceptive thermoreception as well as delayed development of inflammatory hyperalgesia [17]. Likewise, knocking-down of Na_v_1.8 mRNA with antisense oligodeoxynucleotides was effective in alleviating both the inflammatory and neuropathic pain [18-20,37,38]. Also, muO-conotoxin MrVIB, a selective blocker of Na_v_1.8, reduced allodynia and hyperalgesia in neuropathic and chronic inflammatory pain models [39,40]. The present study for the first time revealed that NK-1 activation potentiated Na_v_1.8 currents and shifted both the activation and steady-state inactivation curves of this channel in a hyperpolarizing direction. This change in voltage sensitivity of Na_v_1.8 may decrease the activate threshold and increase the likelihood of action potential firing, and then probably cause a hyperexcitability of the neurons. As shown in Figure 6, the enhancement of excitability was observed in our experiments. Although the involvement of Sar-SP-induced modulation on other ion channels still need to be further investigated, it is assumed that modulation on Na_v_1.8 at least partly contributes to this enhancement of excitability. Similar results were also obtained from studies on modulation of Na_v_1.8 by another peripheral pain-related neuropeptide calcitonin gene related peptide (CGRP) [41] and proinflammatory factors such as 5-hydroxytryptamine and prostaglandin E_2 _[22,42,43]. It is conceivable that the modulation of Na_v_1.8 by NK-1 activation may contribute to peripheral sensitization of pain pathway.

NK-1 receptor is a G-protein coupled receptor [28]. The activation of NK-1 receptors generates various second messengers, which, in turn, trigger a wide range of effector mechanisms underlying regulating cellular excitability and functions [44-47]. In agreement with our previous finding that the modulation of TRPV1 by NK-1 receptor was mediated by activation of PLC and downstream PKC pathway [9], the present results also proved the involvement of PKC in the interaction between NK-1 and Na_v_1.8. As shown in Figure 3 and Figure 4, PKC inhibitor BIM completely blocked Sar-SP-induced potentiation of Na_v_1.8 currents, whereas PKC activator PMA could mimic the effects of Sar-SP on Na_v_1.8 currents. These results suggest that NK-1 modulates Na_v_1.8 in a PKC-dependent pathway. There are many other papers confirmed the enhancement of Na_v_1.8 by PKC pathway [24,48,49]. However, the inconsistent results have been reported. Gold et al. reported that PKC activation also caused an increase in the amplitude of the TTX-resistant current in rat DRG neurons. But this increase was not associated with a shift in the activation curve [23]. Vijayaragavan and colleagues reported that in *Xenopus oocytes *expression system, PKC activator PMA caused a decrease of Na_v_1.8 current and a right shift of the activation curve [29]. The reason for the difference is still unclear.

Furthermore, we observed that PKCε inhibitor εV1-2 completely blocked Sar-SP-induced potentiation of Na_v_1.8 currents, suggesting that PKCε was the main mediator of NK-1 potentiation, in consistence with the modulation of TRPV1 by NK-1 [9].

In addition to the PKC pathway, several reports showed that PKA was also involved in the increase of TTX-resistant currents by proinflammatory agents (5-HT, PGE_2_) [22,23,42]. However, PKA inhibitor H89 failed to prevent Sar-SP-induced potentiation of Na_v_1.8 in the present study. These suggested the diverse mechanisms underlying modulation of Na_v_1.8 by the different proinflammatory agents. Therefore, the modulatory action of NK-1 may be predominately mediated by PKC, particularly by PKCε, but not PKA.

It is well documented an increase in expression of Na_v_1.8 in DRG neurons in several inflammatory pain models [50-53]. Our previous results have revealed that both the NK-1 expression and phosphorylation of PKCε are up-regulated in DRG after CFA-induced inflammation [9,33]. Therefore, we assume that the modulation of Na_v_1.8 by NK-1 via PKCε is likely to be stronger after peripheral inflammation. In support of this view, the present study showed that not only the effect of NK-1 activation on Na_v_1.8 currents was significantly potentiated, but also the rate of Sar-SP-responsive neurons following CFA treatment. It is conceivable that the modulation of Na_v_1.8 by NK-1 may amplify peripheral nociceptive inputs and in turn strengthen activation of the pain-sensitive neurons in the spinal cord, contributing to inflammatory pain.

## Conclusion

Substance P (SP) receptor NK-1 and TTX-resistant sodium channel Na_v_1.8 expressed on nociceptors are two important molecules for pain processing. The present study for the first time investigated their interaction in rat DRG neurons. The results showed that activation of NK-1 receptor potentiates Na_v_1.8 sodium current via PKCε-dependent signaling pathway, probably participating in the generation of inflammatory hyperalgesia.

## Methods

### Animals

Male adult (100–150 g) Sprague-Dawley rats (obtained from the Experimental Animal Center, Shanghai Medical College of Fudan University, China) were used in our experiments. Rats were on a 12 h light/dark cycle with a room temperature of 22 ± 1°C and received food and water *ad libitum*. All experimental procedures were approved by the Shanghai Animal Care and Use Committee and followed the policies issued by the International Association for the Study of Pain on the use of laboratory animals. All efforts were made to minimize animal suffering and reduce the numbers of animals used.

### Cell preparation

Culture of DRG neurons was established as described previously [33]. Briefly, DRGs from L_4_-L_6 _lumbar segments were dissected and incubated at 36.8°C for 25 min in DMEM containing 3 mg/ml collagenase (type IA, Sigma, St. Louis, MO) and, 1 mg/ml trypsin (type I, Sigma). The ganglias were then gently triturated using fine fired-polished Pasteur pipettes. The dissociated DRG neurons were plated onto coverslips (10 mm diameter) in the 3.5 cm culture dishes and incubated with Standard external solution containing (in mM) 150 NaCl, 5 KCl, 2 CaCl_2_, 1 MgCl_2_, 10 HEPES, and 10 glucose, adjusted to pH 7.4 with NaOH.

### Patch-clamp recordings

Whole-cell voltage-clamp and current-clamp recordings of DRG neurons were performed at room temperature (20–22°C) with an EPC-9 amplifier (HEKA Elektronik, Lambrecht/Pfalz, Germany). Stimulation protocols and data acquisition were controlled by the software Pulse and Pulsefit 8.5 (HEKA Elektronik). Neurons were prepared as above, and all recordings were performed within 2–8 h after plating. All of the recordings were made from small-diameter (15–25 μm) DRG neurons. After gigaohm seal formation and membrane disruption, the whole cell capacitance was cancelled and series resistance was compensated (> 80%). Microelectrodes were fabricated from 1.5 mm out diameter borosilicate capillary glass (Sutter Instruments, Novato, CA) by using a P-97 puller (Sutter Instruments, Novato, CA), and had a resistance of 3–5 MΩ. Electrodes were filled with (in mM): 140 CsF, 1 MgCl_2_, 1 EGTA, 2.5 Na_2_ATP, 10 HEPES, pH was adjusted to 7.2 with CsOH. In recording of Na_v_1.8 currents, the external solution contained (in mM): 32 NaCl, 20 TEA-Cl, 105 choline-Cl, 1 MgCl_2_, 1 CaCl_2_, 0.1 CdCl_2_, 10 HEPES, 0.0005 TTX and 10 glucose, adjusted to pH 7.4 with NaOH. The TEA-Cl, CdCl_2_, TTX was used to inhibit endogenous K^+^, Ca^2+^, and TTX-sensitive sodium currents, respectively. In current-clamp recordings, the electrode solution was changed to: 140 KCl, 1 MgCl_2_, 0.5 CaCl_2_, 5 EGTA, 10 HEPES, 2.5 Na_2_ATP, pH was adjusted to 7.2 with KOH. The external solution was changed to: 150 NaCl, 5 KCl, 2.5 CaCl_2_, 1 MgCl_2_, 10 HEPES, pH was adjusted to 7.4 with NaOH.

### Drugs

All the drugs were purchased from Sigma (St. Louis, MO, USA), except that the PKCε inhibitor εV1-2 and its negative control were from Biomol (Plymouth Meeting, PA). All the drugs are prepared on the day of the experiment from stocks kept at -20°C at a concentration at least 1000-fold the working concentration. [Sar^9^, Met(O_2_)^11^]-substance P (Sar-SP) and PMA were applied close to the cells through a ALA-VM8 perfusion system (ALA Scientific Instruments, Westbury, NY). Inhibitors were applied (where appropriate) to the chamber for 30 min before the perfusion of Sar-SP and PMA and existed during the whole recording course.

### Data analysis

Peak sodium current values were converted to conductance values using the equation:* G *= *I*/(*V*_m _- *E*_Na_), where *G *is the conductance, *I *is the peak current amplitude, *V*_m _is the membrane potential, and *E*_Na _is the equilibrium potential value for Na^+^. The Boltzmann equation used to describe the voltage dependence of activation was of the form: *G/G*_*max *_= 1/(1 + *exp *[(*V*_1/2 _- *V*_*m*_)/*k*]), where *G*_max _is the peak conductance, *V*_1/2 _is the potential at half maximal activation, and *k *is the slope factor. Voltage dependence of steady-state inactivation was described by the Boltzmann function: *I*/*I*_*max *_= 1/(1 + *exp *[(*V*-*V*_1/2_)/*k*]), where *I*_max _is the maximal peak current, *V *is the prepulse membrane potential, *V*_1/2 _is the potential at half maximal activation, and *k *is the slope factor.

Data are expressed as means ± standard error of the mean (SEM). Statistical analysis was performed using SigmaStat software (Systat Software, Chicago, IL). Student's t-test or Mann-Whitney analysis was used to assess differences between means from two groups. One-way ANOVA or Kruskal-Wallis one-way ANOVA was used to assess difference among more groups. *P *< 0.05 was considered significant. Curves were plotted and fitted using Origin software (OriginLab Corporation, Northampton, USA).

## Abbreviations

SP: Substance P; DRG: dorsal root ganglion; NK-1: neurokinin-1; TRPV1: transient receptor potential vanilloid 1; TTX: tetrodotoxin; Sar-SP: [Sar^9^, Met(O_2_)^11^]-substance P; PKC: protein kinase C; BIM: bisindolylmaleimide; PKA: protein kinase A; PGE_2_: prostaglandin E_2_; PMA: phorbol 12-myristate 13-acetate; CFA: complete Freund's adjuvant.

## Competing interests

The authors declare that they have no competing interests.

## Authors' contributions

CLC and HZ performed the patch clamp recordings in DRG neurons. YQZ was partially involved in experimental design and guiding. ZQZ is the corresponding author.
